# Herpes Simplex Virus 1 Envelope Cholesterol Facilitates Membrane Fusion

**DOI:** 10.3389/fmicb.2017.02383

**Published:** 2017-12-06

**Authors:** George A. Wudiri, Seth M. Schneider, Anthony V. Nicola

**Affiliations:** Department of Veterinary Microbiology and Pathology, Paul G. Allen School for Global Animal Health, College of Veterinary Medicine, Washington State University, Pullman, WA, United States

**Keywords:** herpesviruses, herpes simplex viruses, HSV-1, viral entry, membrane fusion, cholesterol

## Abstract

Methyl beta-cyclodextrin (MβCD) treatment of herpes simplex virus 1 (HSV-1) reduced envelope cholesterol levels and inhibited viral entry and infectivity in several cell types, regardless of the dependence of entry on endocytosis or low pH. Viral protein composition was similar in MβCD-treated and untreated virions, and ultrastructural analysis by electron microscopy revealed that cholesterol removal did not grossly affect virion structure or integrity. Removal of envelope cholesterol greatly reduced virion fusion activity as measured by fusion-from-without, suggesting that virion cholesterol is critical for the step of membrane fusion. MβCD-treatment of HSV-1 did not reduce viral attachment to the cells nor endocytic uptake of HSV-1 from the cell surface. The pre-fusion form of gB present in the HSV-1 envelope undergoes conformational changes in response to mildly acidic pH. These gB changes occurred independently of envelope cholesterol. Removal of cholesterol compromised virion stability as measured by recovery of infectivity following cycles of freeze-thaw. Taken together, the data suggest that HSV-1 envelope cholesterol is important for viral entry and infectivity due to a critical role in membrane fusion.

## Introduction

Herpesviruses are ubiquitous pathogens that cause significant morbidity and mortality worldwide. The human herpes simplex viruses cause cold sores and sexually transmitted infections. Serious outcomes of HSV infections include neonatal infections, blindness, and disseminated infections of the immunocompromised (Roizman et al., 2013). Herpesvirus entry requires multiple viral and host determinants. Entry of herpes simplex virus type 1 (HSV-1) is initiated by attachment of virions to cell surface glycosaminoglycans (Shukla and Spear, 2001). All enveloped viruses must fuse with host cell membranes to initiate entry and infection (Barrow et al., 2013). In cell types such as human epithelial cells, attached HSV-1 particles undergo endocytosis followed by fusion of the viral envelope with an endosomal membrane (Nicola et al., 2003). Fusion requires the mildly acidic pH of the endosomal milieu in a cell-dependent manner (Nicola, 2016). In other cell types, such as the model Vero cell line, fusion occurs immediately following attachment at the cell surface in a pH-neutral manner (Wittels and Spear, 1991). HSV-1 membrane fusion requires a complex of envelope glycoproteins gD, gH-gL and the core fusion protein gB (Campadelli-Fiume et al., 2012; Krummenacher et al., 2013; Weed and Nicola, 2017).

Herpes simplex virus is encased in a lipid bilayer envelope that is derived from internal membranes of the host cell. Membrane cholesterol is essential for maintaining membrane order and reducing permeability. Cellular cholesterol is critical for many functions, including cell signaling. Roles for cholesterol in the viral membrane are less well understood. Initial HSV-1 infection of Vero cells is dependent on the presence of cholesterol in the virion envelope (Bender et al., 2003). However, the specific step(s) of the entry process that necessitates cholesterol is not known. Chemical treatment of HSV-1 was employed to reduce the cholesterol content of virions and to assess the role of cholesterol levels on distinct steps of entry and infectivity. The results suggest that virion envelope cholesterol promotes HSV-1 fusion, but is dispensable for attachment to cells and uptake by endocytosis. Despite the role of cholesterol in fusion, the known low pH-triggered conformational changes in the fusion protein gB occurred independently of cholesterol.

## Materials and Methods

### Cells and Viruses

Vero cells (American Type Culture Collection, Manassas, VA, United States), HeLa cells, and HEp-2 cells (provided by Stephen Straus, National Institute of Allergy and Infectious Diseases) were propagated in Dulbecco modified Eagle medium (Gibco/Life Technologies, Grand Island, NY, United States) supplemented with 10% fetal bovine serum (Atlanta Biologicals, Flowery Branch, GA, United States). CHO-nectin-1 (M3A) cells (Geraghty et al., 1998), CHO-nectin-2 (M2A) cells (Warner et al., 1998), and B78-nectin-1 (C10) cells (Miller et al., 2001) (provided by Roselyn Eisenberg and Gary Cohen, University of Pennsylvania) are stably transformed with the nectin-1 or nectin-2 gene and contain the *E. coli lacZ* gene under the control of the HSV-1 ICP4 gene promoter. CHO-nectin-1 and CHO-nectin-2 cells were propagated in Ham’s F-12 nutrient mixture (Gibco/Life Technologies) supplemented with 10% fetal bovine serum, 150 μg of puromycin (Sigma–Aldrich, St. Louis, MO, United States)/ml, and 250 μg of G418 sulfate (Thermo Fisher Scientific, Fair Lawn, NJ, United States)/ml. B78-nectin-1 cells were propagated in DMEM supplemented with 5% FBS and 6 μg/ml of puromycin, and 250 μg/ml of G418 sulfate. HSV-1 strain KOS was provided by Priscilla Schaffer, Harvard Medical School, HSV-1 strain ANGpath was provided by Thomas Holland, Wayne State University and HSV-1 KOS-tk12, which contains the *lacZ* gene under the control of the HSV-1 ICP4 gene promoter was provided by Patricia Spear, Northwestern University.

### Virus Preparation

175 cm^2^ flasks of Vero cells were infected with HSV-1 (MOI 0.001) and incubated at 37°C for 3 days. Infected cell supernatant was subjected to centrifugation at 300 × *g* at 4°C for 10 min. Pellet containing cellular debris was discarded. Clarified supernatant containing extracellular virions was pelleted at 27,000 × *g* for 45 min through a 5% sucrose-PBS cushion. Virus pellets were resuspended overnight at 4°C in serum-free medium containing 20 mM HEPES. Concentrated virus was layered onto a 10–30%–60% sucrose-PBS step gradient. Following centrifugation at 16,000 × *g* for 4.5 h, the visible, virion-containing band at the 30–60% interface was collected via side puncture. Following centrifugation at 27,000 × *g* for 45 min, virus was resuspended in serum-free medium containing 20 mM HEPES and stored at -80°C.

### MβCD-Treatment of HSV-1

Herpes simplex virus 1 strain KOS was treated with different concentrations of methyl beta-cyclodextrin (MβCD; Sigma–Aldrich) in serum-free, sodium bicarbonate-free DMEM containing 20 mM HEPES (Gibco/Life Technologies) for 30 min at room temperature. Samples were then diluted 30-fold in serum-free medium and used immediately. The maximum concentration of residual MβCD in diluted virus preparations (0.17 mM) did not inhibit HSV-1 entry into cells (Wudiri et al., 2014).

### Beta-Galactosidase Reporter Assay of HSV-1 Entry

Confluent cell monolayers grown in 96-well dishes were infected with HSV-1 KOS or KOS-tk12 (multiplicity of infection [MOI] of 1) for 6–8 h. 0.5% IGEPAL (Sigma–Aldrich) cell lysates were prepared, chlorophenol red-beta-D-galactopyranoside (Roche Diagnostics, Indianapolis, IN, United states) was added, and the beta-galactosidase activity was read at 595 nm with an ELx808 microtiter plate reader (BioTek Instruments, Winooski, VT, United States). Beta-galactosidase activity indicated successful entry (Delboy et al., 2006). Mean results and standard deviations were calculated for four replicate samples

### Plaque Assay

Herpes simplex virus 1 was titered by limiting dilution. At 18–24 h p.i., culture medium was removed, and cells were fixed with ice-cold methanol-acetone solution (2:1 ratio) for 20 min at -20°C and air-dried. Virus titers were determined by immunoperoxidase staining with anti-HSV polyclonal antibody HR50 (Fitzgerald Industries, Concord, MA, United States).

### Cholesterol Content of HSV-1

The cholesterol content of HSV-1 strain KOS virions was determined using an Amplex Red Cholesterol Assay Kit (Thermo Fisher Scientific) according to the manufacturer’s instructions. Samples were analyzed with a Tecan Infinite M1000PRO plate reader using an excitation wavelength of 560 nm and an emission wavelength of 590 nm.

### Determination of HSV-1 Genome Copy Number by Real-Time PCR

Samples were treated with 2 μg/ml DNase (Bio-Rad) to remove any free HSV-1 DNA that is not associated with viral particles. Viral genomic DNA was extracted using the QIAamp DNA Blood Kit (Qiagen, Germantown, MD, United States). HSV-1 transcripts were quantitated using the CFX96 Real-Time PCR detection system (Bio-Rad). Primers [Integrated DNA Technologies (IDT), Coralville, IA, United States] were based on KOS ICP22 sequence, forward (5′ gag ttt ggg gag ttt g 3′) and reverse (5′ ggc agg cgg tgg aga a 3′) (Komala Sari et al., 2013; Walker et al., 2015). A standard curve for the assay was generated using known quantities of a plasmid containing the HSV-1 ICP22 coding region diluted in glycogen.

### SDS–PAGE and Western Blotting

Herpes simplex virus 1 in Laemmli buffer with 200 mM dithiothreitol was boiled for 5 min. Proteins was resolved by SDS–PAGE on Tris-glycine gels (Thermo Fisher Scientific). For protein staining, the gels were fixed and stained with 0.025% Coomassie brilliant blue (J. T. Baker Chemical Co., Phillipsburg, NJ, United States), 40% methanol (Baker Chemical), and 10% glacial acetic acid (Baker Chemical), followed by destaining with 30% methanol and 7% glacial acetic acid (Delboy et al., 2010). The gel was dried and imaged with a Gel Doc XR imager (Bio-Rad, Hercules, CA, United States). For Western blotting, following transfer to nitrocellulose, membranes were blocked and incubated with HR50, a rabbit polyclonal antibody to HSV-1 strain F. Per the manufacturer, HR50 recognizes HSV-1 late structural proteins, such as the viral envelope glycoproteins. After incubation with horseradish peroxidase-conjugated secondary antibodies, enhanced chemiluminescent substrate (Pierce) was added, and membranes were exposed to X-ray film (Kodak).

### Electron Microscopic Analysis of HSV-1

Purified HSV-1 KOS was mock or MβCD-treated as described and was prepared for negative-stain electron microscopy. Approximately 8 × 10^4^ PFU of virus in 10 μl was added to a formvar/carbon-coated 200 mesh Ni grid (Electron Microscopy Sciences, Hatfield, PA, United States) and stained with 2% aqueous uranyl acetate (Electron Microscopy Sciences). Samples were analyzed with a Tecnai G2 20 Twin transmission electron microscope (Field Emission Instruments Company, Hillsboro, OR, United States) at 200 kV. Images were captured with a 4K Eagle digital camera and processed using Adobe Photoshop CS5.1.

### Fusion-from-without Assay

Confluent Vero cells grown in 24 or 48 well plates were pretreated with 0.5 mM cycloheximide (Sigma) for 15 min at 37°C. HSV-1 ANG path was added (MOI of 20–500) and spinoculated at 200 × *g* at 4°C for 90 min. Cultures were rapidly warmed to 37°C and incubated for 4 h in the continued presence of cycloheximide. Cells were fixed in 100% methanol at -20°C for 20 min and stained with 10% Giemsa solution (Sigma). Micrographs were captured with a Zeiss Axiovert 40C microscope equipped with a Canon PowerShot G6 digital camera. Fusion activity is defined as *a/b × 100*%, where *a* is the number of nuclei sharing a cytoplasm with at least two other nuclei and *b* is the total nuclei. More than 500 nuclei were evaluated per experimental condition.

### HSV-1 Attachment to Cells

The ability of virions to bind to the cell surface was assayed as described previously (Weed et al., 2017). HSV-1 was treated with DNase (Turbo DNA-Free; Thermo Fisher Scientific) according to manufacturer’s instructions. MβCD-treated or mock-treated virions were diluted in ice-cold binding medium [carbonate-free, serum-free DMEM supplemented with 20 mM HEPES and 0.2% bovine serum albumin (BSA)] and added to pre-chilled Vero cells for 1 h on ice. Heparin (2 μg/ml; Sigma) was added to untreated virions as an inhibition of attachment control. Cells were washed twice with ice-cold phosphate-buffered saline (Thermo Fisher Scientific) and cell-associated viral DNA was immediately extracted using the QIAamp DNA Blood Mini Kit (Qiagen) according to the manufacturer’s instructions. HSV-1 genomes were quantitated by real-time PCR.

### Kinetics of HSV-1 Internalization by Endocytosis

Endocytotic uptake of infectious virions from the cell surface was assayed as described previously (Nicola and Straus, 2004). HSV-1 KOS treated with 5 mM MβCD or mock-treated was bound to B78-nectin-1 or CHO-nectin-1 cells on coverslips for 1 h at 4°C. Cells were washed twice with ice-cold PBS and warmed serum-free medium was added. At each time post-infection, non-internalized virus was inactivated with sodium-citrate buffer (pH 3.0) and serum-free medium was added. At 8 h p.i., cells were fixed with methanol and were probed with anti-HSV polyclonal antibody HR50 followed by Alexa Fluor 488-labeled goat anti-rabbit antibody. Cell nuclei were stained with 5 ng/ml of 4′, 6-diamidine-2′-phenylindole dihydrochloride (DAPI; Roche Diagnostics, Indianapolis, IN, United States). Approximately 2000 cells per sample were evaluated with a Leica D4000 epifluorescence microscope. Alexa Fluor 488-positive (infected) and DAPI-stained (total) cells were enumerated, and maximum infectivity was set to 100%.

### Dot Blot Analysis

Herpes simplex virus 1 KOS was diluted in serum-free, bicarbonate-free DMEM with 0.2% BSA and 5 mM (each) HEPES (Life Technologies), 2-(*N*-morpholino) ethanesulfonic acid (MES; Sigma), and sodium succinate (Sigma) to achieve a final pH of 7.4 or 5.1. Samples were incubated at 37°C for 10 min. Samples either were blotted directly to nitrocellulose with a Minifold dot blot system (Whatman) or were first neutralized to pH 7.4 by addition of pretitrated amounts of 0.05 N NaOH. Membranes were blocked and incubated with anti-gB monoclonal antibodies H126, H1359 (Virusys), or SS55 (Bender et al., 2005) provided by G. Cohen and R. Eisenberg, University of Pennsylvania. After incubation with horseradish peroxidase-conjugated secondary antibodies, enhanced chemiluminescent substrate (Pierce) was added, and blots were exposed to X-ray film (Kodak).

### Analysis of gB Oligomeric Structure by PAGE

Herpes simplex virus 1 KOS was diluted in medium as described above for dot blotting. Samples were adjusted to pH 7.4 or 5.1 with pretitrated amounts of 0.05 N HCl and incubated at 37°C for 10 min. 1% sodium dodecyl sulfate (SDS) was added, or samples remained untreated. Polyacrylamide gel electrophoresis (PAGE) sample buffer containing 0.2% SDS and no reducing agent was added (“native” conditions), and proteins were resolved by PAGE. After transfer to nitrocellulose, membranes were blocked and incubated with anti-gB MAb H1359. After incubation with horseradish peroxidase-conjugated secondary antibodies, enhanced chemiluminescent substrate (Pierce) was added, and membranes were exposed to X-ray film (Kodak).

### Annexin V Treatment of HSV-1

Soluble annexin V (Thermo Fisher) or BSA was added to HSV-1 for 1 h at 37°C. Treated virus was pelleted through a 5% sucrose-PBS cushion at 27,000 × *g* at 4°C for 45 min to rid excess protein. Virus preparations were resuspended in serum-free medium supplemented with 0.05% BSA and added to the indicated cell types. Viral entry was measured by beta-galactosidase reporter assay at 6–8 h post-infection.

### Determination of Virion Stability

Methyl beta-cyclodextrin-treated or mock-treated HSV-1 (∼9 × 10^6^ PFU) in serum-free DMEM containing 0.05% BSA was rapidly frozen in a dry ice – ethanol bath, and then thawed on ice. Viral titer was determined immediately or following each of three additional freeze-thaw cycles. Titers are shown relative to the first freeze-thaw, which was set to 100%.

## Results

### Virion Envelope Cholesterol Facilitates HSV-1 Entry via Low pH or pH-Neutral Mechanisms

**FIGURE 1:**
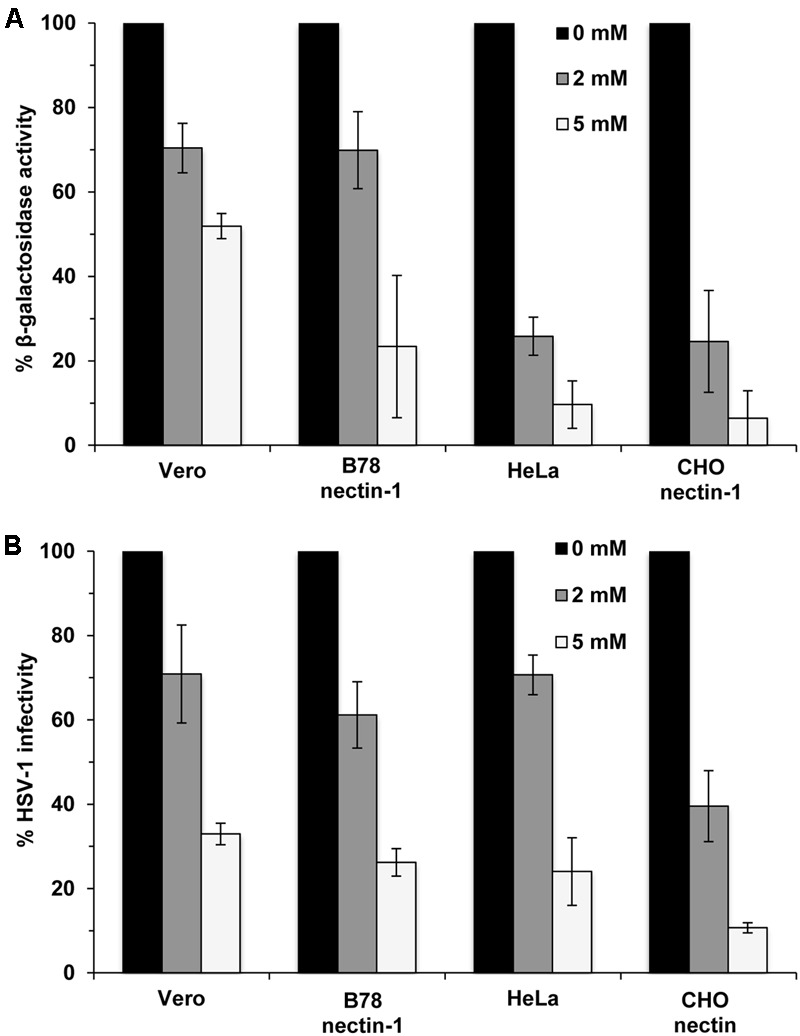
Dependence of viral entry on herpes simplex virus 1 (HSV-1) envelope cholesterol, regardless of the entry route supported by the target cell. **(A)** MβCD-treated or mock-treated (0 mM) HSV-1 was added to cells for 6–8 hr. HSV-1 KOS-tk12 (*lacZ^+^*) was added to Vero and HeLa cells. HSV-1 KOS was added to *lacZ^+^* cell lines B78-nectin-1 and CHO-nectin-1. The percent beta-galactosidase activity relative to that obtained with mock-treated virus is indicated. **(B)** Mock-treated (0 mM) HSV-1 (100 PFU) or MβCD-treated HSV-1 was added to cells for 24 h and infectivity was determined by plaque assay. The results shown are representative of three independent experiments.

Methyl beta-cyclodextrin treatment of HSV-1 inhibits viral entry into Vero cells (Bender et al., 2003). HSV entry can occur by endocytotic or non-endocytotic mechanisms depending on the cell type. Cholesterol might play a differential role in entry depending on the HSV entry route or cell type. To address this possibility, we assessed the effect of cholesterol reduction on entry into cells that support different routes of HSV-1 entry (Nicola et al., 2003, 2005; Nicola and Straus, 2004; Milne et al., 2005). Virions were MβCD-treated or mock-treated and then diluted in serum-free medium prior to infecting Vero cells (pH-neutral, direct penetration at the plasma membrane), B78-nectin-1 cells (pH-neutral endocytosis), HeLa or CHO-nectin-1 cells (low pH-dependent endocytosis). MβCD treatment of HSV-1 inhibited viral entry in a concentration-dependent manner as measured by β-galactosidase activity in all cells tested (**Figure 1A**). HSV-1 infectivity measured by plaque formation was also inhibited in an MβCD-concentration-dependent manner (**Figure 1B**). The data suggest that HSV-1 entry and infectivity is cholesterol-dependent regardless of the entry route.

### Characterization and Ultrastructural Analysis of Cholesterol-Depleted HSV-1

**FIGURE 2:**
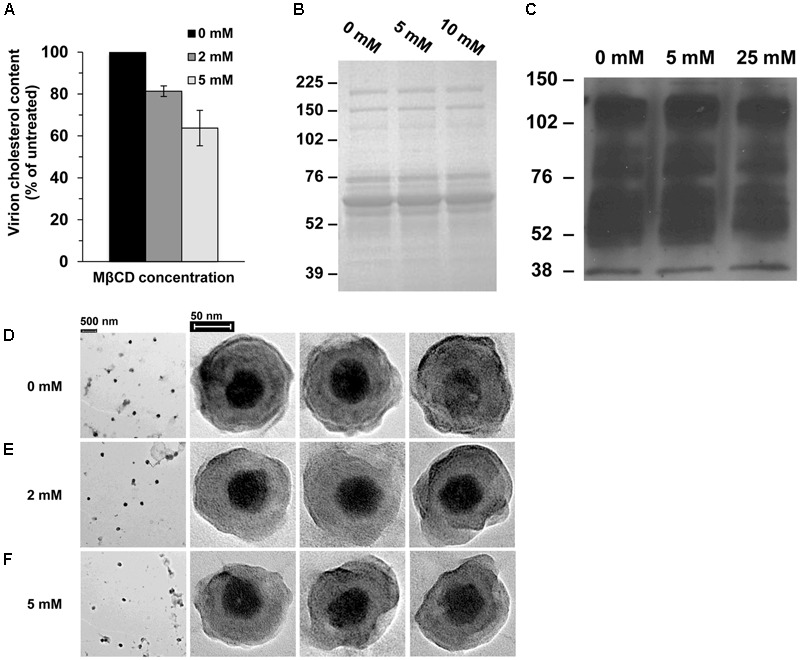
Characterization of cholesterol-reduced HSV-1 particles. **(A)** Cholesterol content of MβCD-treated or mock-treated HSV-1 was measured with the Amplex red cholesterol assay kit. Data are the means of triplicate determinations with standard deviation. **(B,C)** MβCD-treated or mock-treated HSV-1 was separated by SDS–PAGE followed by **(B)** protein staining with Coomassie blue or **(C)** Western blot with anti-HSV polyclonal antibody HR50. Molecular weight standards are indicated to the left in kilodaltons. **(D–F)** Effect of cholesterol on HSV-1 morphology. Negative-stain electron microscopic images of **(D)** mock-treated or **(E,F)** MβCD-treated HSV-1.

To confirm the reduction of viral cholesterol following MβCD-treatment, cholesterol levels were measured by the Amplex red assay. HSV-1 cholesterol levels were reduced by up to 37% when treated with 5 mM MβCD (**Figure 2A**). MβCD-treated HSV-1 had similar protein content and protein levels relative to mock-treated virus (**Figures 2B,C**). The entry defect of cholesterol-reduced HSV-1 (**Figure 1**) might be explained by a global impact on virion morphology. To address this possibility, MβCD-treated virions were subjected to negative-staining transmission electron microscopy. There was no evidence of aggregation of MβCD-treated HSV-1 relative to untreated (**Figures 2D–F**, first column). Cholesterol-reduced virions had similar physical features and similar size as mock-treated HSV-1.

### Envelope Cholesterol Is Critical for HSV-Mediated Fusion with Target Membranes

**FIGURE 3:**
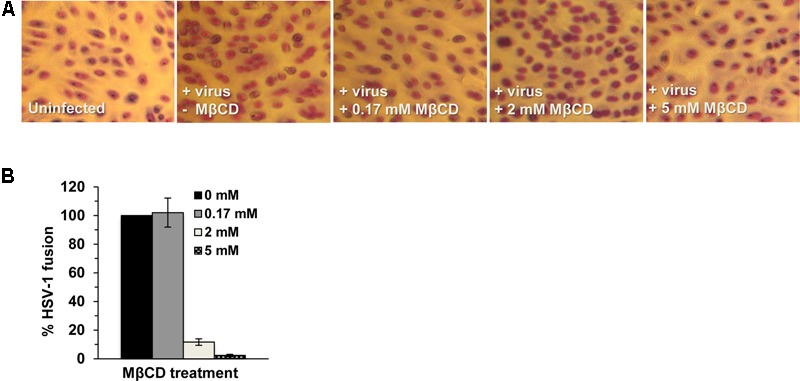
Envelope cholesterol is critical for HSV-1 mediated cell fusion. MβCD-treated or mock-treated HSV-1 ANGpath was diluted in culture medium and added to Vero cells for 4 h in the presence of cycloheximide. Cells were fixed and stained. Micrographs of fusion-from-without (FFWO) were captured **(A)** and quantitated **(B)** as described in Section “Materials and Methods.” The results shown are representative of three independent experiments.

Fusion of the viral envelope with a target host cell membrane is a critical step of entry that results in penetration of the HSV capsid into the cytoplasm. Since fusion during entry is difficult to measure directly, the ability of HSV-1 to mediate virion-induced cell fusion or fusion-from-without (FFWO) has been used as a surrogate assay (Delboy et al., 2008; Roller et al., 2008; Wudiri et al., 2014; Weed et al., 2017). To assess whether cholesterol in the viral envelope is important for HSV-1 fusion, we tested the effect of MβCD-treatment of HSV-1 ANGpath strain on virus-mediated cell fusion (FFWO) (Falke et al., 1985). MβCD-treatment of FFWO strain of HSV-1 ANG path greatly inhibited its fusion activity in a concentration-dependent manner (**Figure 3**). Up to 97% of virion FFWO activity was blocked by treatment with 5 mM MβCD (**Figure 3B**) suggesting that HSV-1 envelope cholesterol is critical for membrane fusion. For these experiments, virions are treated with MβCD and then the mixture is diluted prior to adding to cells. Control treatment of virus with the maximum residual concentration of MβCD after dilution (0.17 mM) resulted in fusion activity similar to mock-treated virus (**Figure 3**).

### HSV-1 Attachment to Target Cells Is Independent of Envelope Cholesterol

**FIGURE 4:**
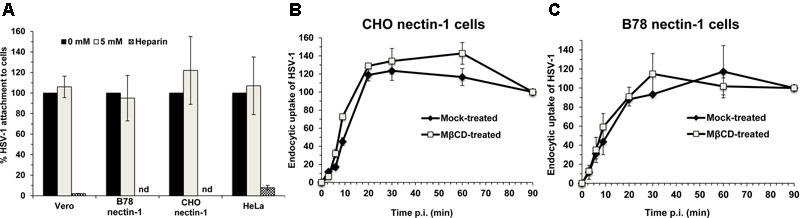
Effect of MβCD-treatment of HSV-1 on cell attachment and on the kinetics of endocytotic internalization. 5 mM MβCD-treated or mock-treated HSV-1 was added to the indicated cells (MOI) on ice for 1 h. **(A)** Cells were rinsed twice with cold PBS, and cell-associated virions were quantitated by qPCR. Data are expressed as % of untreated virus that successfully attached. nd, not determined. **(B,C)** To determine the kinetics of endocytosis of HSV-1, cells were washed and incubated at 37°C, and extracellular virus was inactivated by acid treatment at the indicated times. At 8 h p.i., cells were fixed and fields of ∼2000 cells per sample were evaluated. Total cell number was evaluated by nuclear staining with 4′, 6-diamidine-2′-phenylindole dihydrochloride (DAPI), and infected cells were detected by immunofluorescence with an anti-HSV polyclonal antibody. Infectivity at 90 min p.i., was set to 100%. Data are means of triplicate determinations with standard deviation. The results shown are representative of at least two independent experiments.

We next assessed the effect of envelope cholesterol on HSV-1 attachment to cells. MβCD-treated HSV-1 or mock-treated virus was added to Vero, B78-nectin-1, CHO-nectin-1 or HeLa cells on ice for 1 h at 4°C. Cell-attached HSV-1 was quantitated by qPCR. MβCD-treated HSV-1 attached to all cells in a manner similar to mock-treated HSV-1 (**Figure 4A**). Control soluble heparin inhibited HSV-1 attachment to cells by >90%. These results suggest that the reduced fusion and entry activities of cholesterol-reduced HSV-1 are not due to a defect in HSV-1 attachment.

### Endocytotic Internalization of Infectious HSV-1 Occurs Independently of Envelope Cholesterol

Endocytosis of viral particles depends on the initial interaction of the viral envelope with the host cell. Thus, HSV-1 envelope cholesterol may play a role in viral uptake by endocytosis in addition to a role in fusion. To measure specifically the rate of uptake of infectious particles, MβCD-treated HSV-1 or mock-treated virus was first bound to CHO-nectin-1 cells or B78-nectin-1 cells at 4°C. At various times after warming of cultures to 37°C, extracellular virus was inactivated. Cultures were incubated for a total of 8 h, and cells that expressed newly synthesized viral antigen were quantitated by immunofluorescence microscopy. The acquisition of viral resistance to inactivation, which reflects uptake by endocytosis, was similar for MβCD-treated HSV-1 and mock-treated HSV-1 over 90 min of infection of both cell types (**Figures 4B,C**). HSV-1 was endocytosed rapidly into CHO-nectin-1 cells and B78-nectin-1 with a half-time of ∼9 min (**Figures 4B,C**), consistent with previous reports (Nicola and Straus, 2004). The data suggest that the endocytotic internalization of infectious HSV-1 is not a step in viral entry that requires envelope cholesterol.

### Low pH-Induced Conformational Changes in gB Occur Independently of Envelope Cholesterol

**FIGURE 5:**
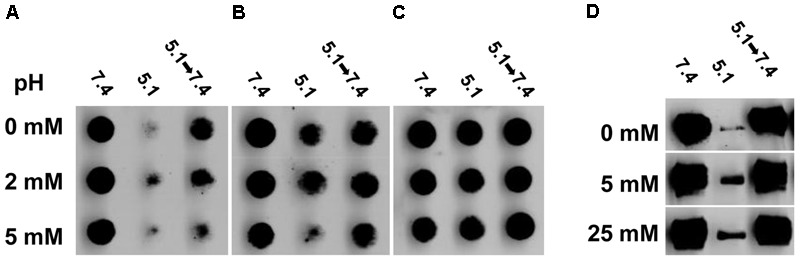
Envelope cholesterol is not required for low pH-triggered conformational changes in gB. MβCD-treated or mock-treated HSV-1 were treated with medium at pH 7.4, or 5.1, or 5.1 and then neutralized back to 7.4. **(A–C)** Virus was directly blotted onto nitrocellulose membranes and probed at neutral pH with monoclonal antibody **(A)** H126 to gB domain I, **(B)** SS55 to gB domain V, or **(C)** H1359 to gB domain III. **(D)** Samples were treated with 1% SDS and separated by “native” PAGE. Western blots were probed with monoclonal antibody H1359 to gB.

Herpes simplex virus glycoprotein gB is a trimeric class III fusion protein that undergoes reversible conformational changes. Upon exposure to mildly acidic pH such as that present in an endosomal pathway, gB becomes more hydrophobic, undergoes a shift in tryptophan fluorescence, has detectable antigenic changes in Domains I and V, and shifts to a lower density oligomeric form (Dollery et al., 2010, 2011; Siekavizza-Robles et al., 2010; Stampfer et al., 2010; Weed et al., 2017). Since our results are consistent with a role for cholesterol in membrane fusion, we theorized that cholesterol would be important for fusion-associated conformational changes in gB. Mock-treated HSV-1 gB exposed to the mildly acidic pH of 5.1 had reduced reactivity with monoclonal antibodies H126 (domain I; **Figure 5A**, top row) and SS55 (domain V; **Figure 5B**, top row). This is indication of conformational change in the respective domains. When HSV-1 is exposed to pH 5.1 and then neutralized back to pH 7.4, the reactivity of these antibodies is restored, denoting reversibility of the changes (**Figures 5A,B**, top row). In contrast, the reactivity of H1359 (domain III) with HSV-1 treated with pH 7.4 or 5.1 remains unchanged, suggesting that the change in gB is not global, consistent with previous reports (**Figure 5C**; Dollery et al., 2010). When HSV-1 was first treated with 2 mM (middle row) or 5 mM (bottom row) MβCD, low pH-triggered changes in the H126 (**Figure 5A**) and SS55 (**Figure 5B**) epitopes were similar to those detected in mock-treated HSV-1 gB.

Herpes simplex virus 1 gB oligomer also undergoes a change when exposed to pH ≤ 6.2 and 1% SDS with a loss of the higher molecular weight species (**Figure 5D**, top row; Dollery et al., 2010). These bands are restored when HSV-1 gB is neutralized back to pH 7.4 (**Figure 5D**), reflecting reversibility of the gB changes. 5 mM MβCD-treated HSV-1 underwent a low pH-triggered, reversible change in the gB oligomer comparable to mock-treated virus (**Figure 5D**, bottom row). Treating HSV-1 with up to 25 mM MβCD did not inhibit the pH-induced oligomeric change (**Figure 5D**, bottom row). Taken together, the data suggest that although HSV-1 fusion is highly dependent on envelope cholesterol, the low pH triggered conformational changes that occur in HSV-1 gB are independent of envelope cholesterol.

### Effect of Soluble Annexin V on HSV-1 Entry by Diverse Entry Pathways

**FIGURE 6:**
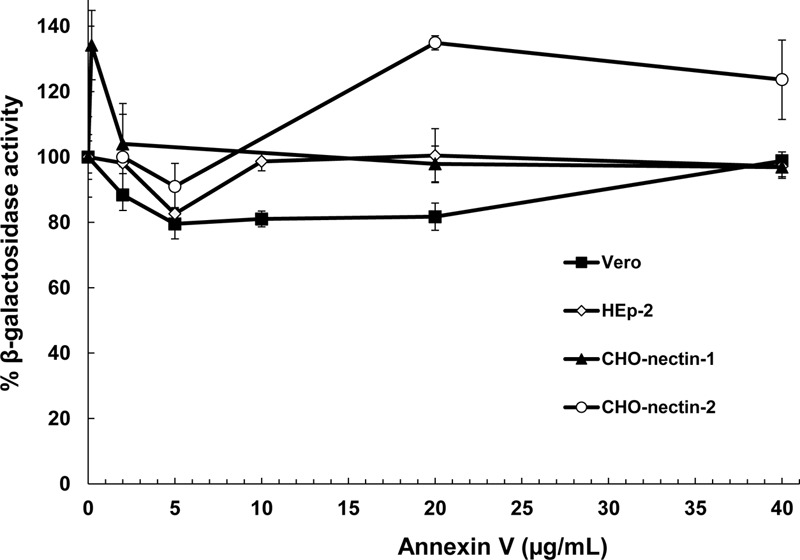
Effect of a ligand for phosphatidylserine (PS), an HSV-1 envelope lipid constituent, on viral entry. Soluble annexin V or BSA was added to preparations of different strains of HSV-1 for 1 h at 37°C. Virus was then pelleted to remove unbound annexin. Treated HSV-1 KOS was added to CHO-nectin-1 cells, KOS-tk12 was added to Vero or HEp-2 cells, and ANG path was added to CHO-nectin-2 cells. At 6 to 8 h post-infection, beta-galactosidase activity of infected cell lysates was determined as an indicator of viral entry. Data are means of quadruplicate determinations with standard deviation.

In addition to cholesterol, the HSV-1 envelope contains the phospholipid, phosphatidylserine (PS) (Asher et al., 1969). An emerging concept in viral entry is that many DNA and RNA viruses including vaccinia, baculovirus, Sindbis, Ebola, and dengue viruses contain PS in the outer leaflet of the viral envelope, and engage PS receptors on the host cell surface to initiate endocytosis and entry (Moller-Tank and Maury, 2014). Apoptotic cells overexpress PS in their plasma membranes, triggering their internalization. Viruses mimic apoptotic cells in this regard. Inhibition by annexin V, a PS-binding molecule, is an indicator that viral envelope PS is important for viral entry (Callahan et al., 2003; Mercer and Helenius, 2008). HSV-1 was incubated with soluble annexin V and tested for entry into cells that support known endocytotic or non-endocytotic entry routes for HSV. HSV-1 KOS or its *lacZ*^+^ derivative KOS-tk12 enters Vero and HEp-2 cells by direct penetration. KOS enters CHO-nectin-1 cells by a low pH-dependent route, and HSV-1 ANG path enters CHO-nectin-2 cells by a pH-neutral pathway. Annexin V pretreatment of HSV-1 had little to no effect on entry into Vero (Meertens et al., 2012), HEp-2, CHO-nectin-1, or CHO-nectin-2 cells (**Figure 6**). Pretreatment with control BSA yielded a similar effect on HSV-1 entry. These results suggest that PS is not critical for HSV entry, regardless of the cellular entry requirement of endocytosis or endosomal low pH.

### Cholesterol Is Critical for HSV-1 Virion Stability

**FIGURE 7:**
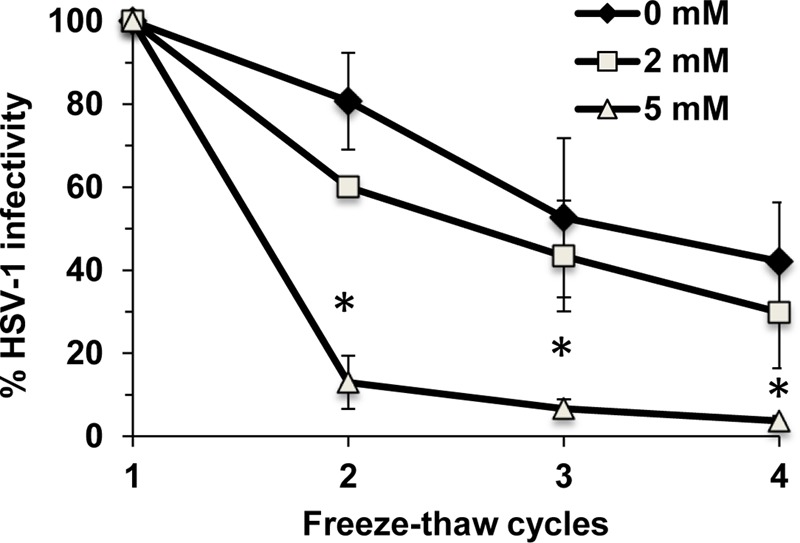
Envelope cholesterol is critical for the stability of infectious HSV-1 particles. MβCD-treated or mock-treated HSV-1 was subjected to up to four freeze-thaw cycles, and infectivity was determined by plaque assay after each thaw. Data are the mean of triplicate determinations with standard deviation. Student’s *t-*test (0 mM vs. 5 mM). ^∗^*p* ≤ 0.01.

Cholesterol plays a key role in maintaining the integrity and stability of cellular membranes. It may also help maintain the stability of HSV-1. ∼9 × 10^6^ PFU of HSV-1 was subjected to repeated freeze-thaw cycles. Viral infectivity was determined after each thaw. The infectivity of mock-treated HSV-1 was reduced by 20% after the second thaw and by 58% after the fourth thaw (**Figure 7**). In contrast, the infectivity of HSV-1 treated with 5 mM MβCD was reduced by 87% after only the second thaw and was almost completely abolished (reduced by 96%) after the fourth thaw (**Figure 7**). Taken together, the results suggest that envelope cholesterol is vital for the stability and infectivity of HSV-1.

## Discussion

Viral membrane cholesterol facilitates the entry and infectivity of many enveloped viruses with some exceptions (Campbell et al., 2002; Guyader et al., 2002; Lu et al., 2002; Bender et al., 2003; Brown and Lyles, 2003; Huang et al., 2006; Bremer et al., 2009; Gudleski-O’Regan et al., 2012). Here, we use MBCD-treated virions to demonstrate that cholesterol is critical for HSV-1 membrane fusion using a FFWO approach. HSV-1 propagated on cells containing the cholesterol precursor desmosterol was previously shown to be cholesterol-free, yet infectious (Wudiri and Nicola, 2017). This suggests that the function(s) of cholesterol in the HSV-1 envelope, such as its role in fusion, can be performed by other sterols. Cholesterol was not required for HSV-1 attachment to cells or for virus uptake from the plasma membrane by endocytosis. Cholesterol present in the host membranes is also important for HSV-1 membrane fusion (Wudiri et al., 2014). This contrasts with influenza membrane fusion, which only requires viral envelope cholesterol (Sun and Whittaker, 2003).

The role of cholesterol in membrane fusion including virus-mediated fusion has been studied for many years (White and Helenius, 1980; Young et al., 1983; Eidelman et al., 1984). Envelope cholesterol participates specifically in the membrane fusion function of several viruses (Daya et al., 1988; Viard et al., 2002; Sun and Whittaker, 2003). How might cholesterol impact HSV-1 fusion? Conformational changes in gB are critical for HSV-1 membrane fusion. Interestingly, HSV envelope cholesterol is not required for the low pH-induced conformational changes in the antigenic and oligomeric structures of gB examined here, nor is it required for the reversibility of these changes (**Figure 5**). It remains to be seen whether HSV-1 envelope cholesterol impacts the roles of gD and gH/gL in entry and fusion. During membrane fusion, cholesterol may promote viral membrane curvature and bending necessary for the merging of juxtaposed membranes. Human cytomegalovirus upregulates expression of host LDL receptor-related protein 1 early in infection, resulting in progeny virions that have reduced cholesterol and reduced infectivity (Gudleski-O’Regan et al., 2012).

Viral cholesterol removal negatively impacted HSV-1 entry regardless of whether entry proceeds by endocytosis or whether entry requires endosomal pH. Envelope cholesterol may serve a conserved function(s) in low pH-dependent and pH neutral fusion mechanisms. Replenishing MβCD-treated influenza and hepatitis B virions with exogenous cholesterol restores infectivity (Sun and Whittaker, 2003; Bremer et al., 2009). These viruses obtain their envelopes from the plasma membrane. HSV-1 derives its envelope from internal membranes, which tend to be less cholesterol-rich. It remains to be determined whether cholesterol-depleted HSV-1 can be successfully replenished. We also provide evidence that viral cholesterol greatly influences the stability of infectivity of HSV-1. Cholesterol may help maintain the integrity of the viral envelope allowing the particle to remain infectious for extended periods.

## Author Contributions

Conceived and designed the experiments: GW and AN. Performed the experiments: GW. Analyzed the data: GW, SS, and AN. Wrote the paper: GW, SS, and AN.

## Conflict of Interest Statement

The authors declare that the research was conducted in the absence of any commercial or financial relationships that could be construed as a potential conflict of interest.
